# The combined focal loss and dice loss function improves the segmentation of beta-sheets in medium-resolution cryo-electron-microscopy density maps

**DOI:** 10.1093/bioadv/vbae169

**Published:** 2024-11-22

**Authors:** Yongcheng Mu, Thu Nguyen, Bryan Hawickhorst, Willy Wriggers, Jiangwen Sun, Jing He

**Affiliations:** Department of Computer Science, Old Dominion University, Norfolk, VA 23529, United States; Department of Computer Science, Old Dominion University, Norfolk, VA 23529, United States; Department of Computer Science, Old Dominion University, Norfolk, VA 23529, United States; Department of Mechanical and Aerospace Engineering, Old Dominion University, Norfolk, VA 23529, United States; Department of Computer Science, Old Dominion University, Norfolk, VA 23529, United States; Department of Computer Science, Old Dominion University, Norfolk, VA 23529, United States

## Abstract

**Summary:**

Although multiple neural networks have been proposed for detecting secondary structures from medium-resolution (5–10 Å) cryo-electron microscopy (cryo-EM) maps, the loss functions used in the existing deep learning networks are primarily based on cross-entropy loss, which is known to be sensitive to class imbalances. We investigated five loss functions: cross-entropy, Focal loss, Dice loss, and two combined loss functions. Using a U-Net architecture in our DeepSSETracer method and a dataset composed of 1355 box-cropped atomic-structure/density-map pairs, we found that a newly designed loss function that combines Focal loss and Dice loss provides the best overall detection accuracy for secondary structures. For β-sheet voxels, which are generally much harder to detect than helix voxels, the combined loss function achieved a significant improvement (an 8.8% increase in the F_1_ score) compared to the cross-entropy loss function and a noticeable improvement from the Dice loss function. This study demonstrates the potential for designing more effective loss functions for hard cases in the segmentation of secondary structures. The newly trained model was incorporated into DeepSSETracer 1.1 for the segmentation of protein secondary structures in medium-resolution cryo-EM map components. DeepSSETracer can be integrated into ChimeraX, a popular molecular visualization software.

**Availability and implementation:**

https://www.cs.odu.edu/~bioinfo/B2I_Tools/.

## 1 Introduction

A cryo-electron microscopy (cryo-EM) map is a 3D image reconstructed from multiple 2D images of biological specimens that were imaged in their native, frozen environment. Cryo-EM maps are often the terminal results of structural biology studies and are therefore deposited into the Electron Microscopy Data Bank (EMDB) (Lawson *et al.* 2016), which allows for their subsequent interpretation and validation by a reasonably informed public. In cryo-EM density maps, the intensity value at each voxel approximates the local density of the biomolecules. The maps are 3D images that can be used to determine their atomic structures. However, the relative ease of cryo-EM map interpretation hides many details of the production of such maps, such as sample preparation, image formation physics, and extensive computational processing.

With the advancement of detector technology in the last decade, the attainable resolution of cryo-EM maps has reached 2–4 Å. At such a high resolution, details of the backbone and amino acid side chains can be resolved, and atomic structures can be derived. Consequently, cryo-EM has become a dominant technology for solving atomic resolutions for many molecular complexes (Zhang *et al.* 2020, Fromm *et al.* 2023). The atomic structure interpretations are typically deposited into the Protein Data Bank (PDB) (Berman *et al.* 2000) and linked with the EMDB entry (EMD). However, cryo-EM density maps are still commonly solved at medium resolution (5–10 Å) in some local regions or globally, which complicates their structural interpretation. Moreover, in cryo-electron tomography (Frank 2006), which is normally a low-resolution imaging technique, density maps increasingly reach medium resolution due to advancements in subtomogram averaging (Heumann *et al.* 2011). At medium resolution, amino acid side chains are indistinguishable, and the backbone of the protein chain is difficult to recognize. Without known atomic structure templates, building reasonably accurate atomic models from medium-resolution maps is rarely possible. However, when a template structure is available in the PDB, an atomic model can be derived via fitting (Chan *et al.* 2012, Kovacs *et al.* 2018). When no suitable template is available, possible configurations of the backbone can be suggested by graph algorithms to optimize the secondary-structure elements that are detected from the cryo-EM map and those predicted from the amino acid sequence (Abeysinghe *et al.* 2008, Al Nasr *et al.* 2014, Biswas *et al.* 2017).

**Figure 1.**
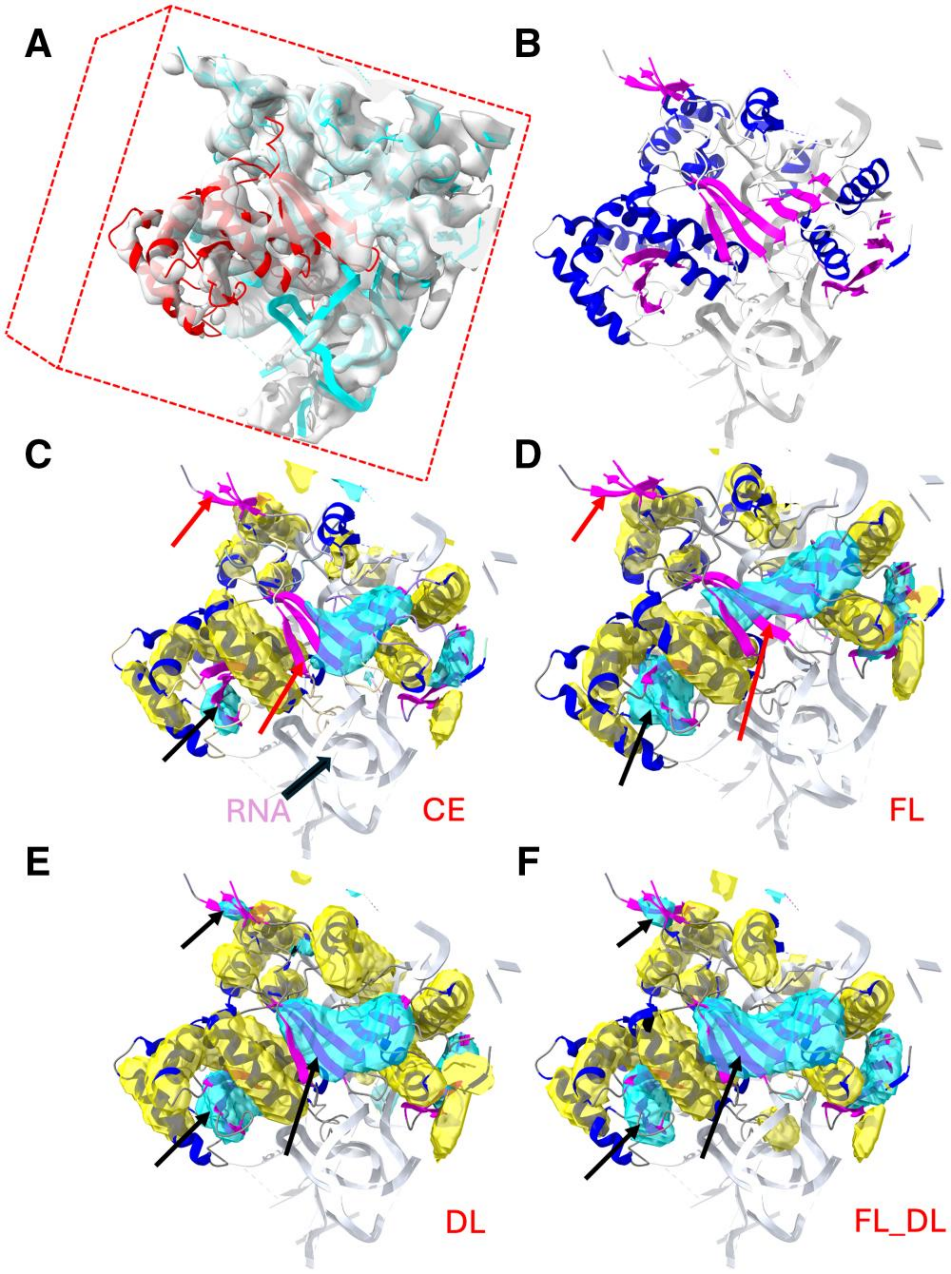
Helix and β-sheet regions segmented from a box-cropped cryo-EM density map using different models trained with different loss functions. (A) The box-cropped cryo-EM density map EMD-2620, EMDB ID, (transparent gray) superimposed on the corresponding atomic structure (ribbon) around chain BH of PDB-4UJE (red). (B) Helices (blue ribbon), β-sheets (magenta ribbon), and the remaining molecular segments (gray) of the atomic structure of the box-cropped region. (C) to (F) show the helix regions (yellow) and β-sheet regions (cyan) that were segmented using the loss functions CE, FL, DL, and FL_DL, respectively, superimposed on the atomic structure helix (blue ribbon) and β-sheet (magenta). The red arrows indicate the β-sheet regions that were not detected using CE and FL, and the black arrows indicate the β-sheets that were better detected using DL and FL_DL. All of the molecular graphics in the figures were created using the ChimeraX software (Pettersen *et al.* 2021).

Secondary structures, such as α-helices and β-sheets, are major building blocks of a protein, and their relative location in the 3D volume of a density map provides constraints for fitting templates or for tracing the backbone of a protein structure. To examine a component of a medium-resolution cryo-EM map, Fig. 1A shows a box-cropped region (shaded transparent gray) and its corresponding atomic structure. A box-cropped region often includes a protein chain (red ribbon) and its partial neighboring chains (cyan ribbon). Helices (blue) and β-sheets (magenta) of the multiple chains in the box-cropped region are shown in Fig. 1B. Secondary structure segmentation, which we address in this work, is an approach to detect helix regions and β-sheet regions in a medium-resolution map. Figure 1C–F illustrates the helix (yellow) and β-sheet (cyan) regions detected using the secondary-structure segmentation approach explored in this study.

Multiple image-processing methods for detecting secondary structures in medium-resolution cryo-EM maps have been developed over the years (Jiang *et al.* 2001, Kong and Ma 2003, Dal Palu *et al.* 2006, Baker *et al.* 2007, Zeyun and Bajaj 2008, Rusu and Wriggers 2012, Si and He 2013). Recent deep learning (DL) approaches show much promise (Li *et al.* 2016, He and Huang 2021, Mu *et al.* 2021, Wang *et al.* 2021). The remaining problem in the discipline that is hardest to solve is how to accurately detect β-sheet regions. This is because alpha helices are readily visible at medium resolution, but individual β-strands require 5 Å resolution or better. When strands combine into β-sheets, they have less distinguishable shapes than helices, and they typically exhibit lower density and less predictable shapes and sizes. In some cases, the dense core of a β-sheet may appear cylindrical, due to which the β-sheet may be mistaken for a helix. In a recent statistical analysis, an average residue-level F_1_ score of 72% was obtained for the detection of helices using DeepSSETracer, but only a 65% F_1_ score was obtained for the detection of β-sheets (Mu *et al.* 2021). When a different method, Emap2sec+ (Wang *et al.* 2021), was used (Mu *et al.* 2021), a lower F_1_ score for β-sheet detection was also observed (42% versus 77% for helix).

Although several DL methods have been proposed for secondary structure segmentation in cryo-EM maps, how the detection performance is affected by important network elements has not yet been systematically studied. A critically important measure of such performance is the neural network loss function because it determines how well the network models the training data. Some loss function designs can be reasonably expected to provide better learning outcomes than others, depending on the application. Cross-entropy (CE) is a popular loss function used in many learning problems, including in secondary structure segmentation with EMap2sec+ and EMNUSS (He and Huang 2021, Wang *et al.* 2021). However, CE is known for its weakness in handling class imbalance (Lin *et al.* 2017). A model trained with CE tends to perform poorly in classes with fewer voxels in the training data, even though the overall accuracy of CE can be relatively good. Dice loss (DL) is another function that is popularly used in medical imaging problems (Zhao *et al.* 2020). Compared to CE, the Dice coefficient is less sensitive to class imbalance (especially to any imbalance between foreground and background voxels), but it is less smooth and, consequently, difficult to optimize. In previous studies on image segmentation, CE and DL have been individually used as the optimization objective for a U-Net-like fully convolutional neural network (CNN) (Ronneberger *et al.* 2015, Çiçek *et al.* 2016, Milletari *et al.* 2016). An alternative, Focal loss (FL), was recently proposed by Lin *et al.* (Lin *et al.* 2017). FL is designed to downweight the loss from easy voxels, which can be predicted more confidently. The focal concept can be similarly adapted to DL in an approach termed *focal DL* (Prencipe *et al.* 2022). Alternatively, FL can be combined directly with DL, as implemented in this article.

Just as in visual recognition, we expect to be able to recognize different types of patterns with varying levels of difficulty. For example, noise voxels that are unrelated to molecular density are generally easiest to distinguish, followed by helix regions due to their prominent cylindrical shape, whereas the irregularly shaped β-sheets and loops are more difficult to detect. In addition, the presence of helices and β-sheets as fractions of volume varies from map to map, depending on the secondary structure of the corresponding protein chain(s). These varying levels of difficulty in recognizing different types of patterns and the abundance of patterns in the training data pose challenges to the design of a loss function that performs well across all patterns. In this study, we utilized our previous U-Net like architecture in DeepSSETracer (Mu *et al.* 2021), but the work particularly focuses on the design and validation of different loss functions.

In this article, we describe our extensive study of the performance of five loss functions and we provide insights for the winning loss function using specific examples. Compared to our preliminary study of loss functions (Mu *et al.* 2022), our current study utilized a different dataset with a much larger amount of data and offers a trained model that is more robust and general for end users. The model has been incorporated into DeepSSETracer (version 1.1), which is bundled with the popular molecular viewer ChimeraX. Using a box-cropped cryo-EM density dataset, we show that FL_DL is effective in improving the detection of β-sheets that are generally harder to detect correctly than helices and less abundant in the dataset.

## 2 Methods

In this section, we describe the development of our dataset, the five loss functions, the architecture, and the training process of our CNN.

### 2.1 Dataset preparation

Our dataset consisted of 1355 map/structure pairs of cryo-EM density maps cropped to their corresponding atomic structures. The dataset is a variant of a previously developed dataset with an additional box-cropping adaptation. In our earlier study (Mu *et al.* 2021), we developed a method of selecting protein chains from EMDB-deposited medium-resolution cryo-EM maps. This chain-based method uses the atomic (PDB-deposited) structure of an individual chain to mask the corresponding density region in the cryo-EM map. To reduce the bias toward repetitive chains in the PDB, chains that shared over 70% sequence identity in the same PDB entry were removed. To eliminate low-quality map/structure pairs, each map/structure match was screened using both the averaged helix cylindrical similarity score and the difference between the precision and the recall (Sazzed *et al.* 2020, Nguyen *et al.* 2023). Four bins with decreasing quality were created, and the dataset of 1355 pairs in the top three quality bins was used.

**Figure 2.**
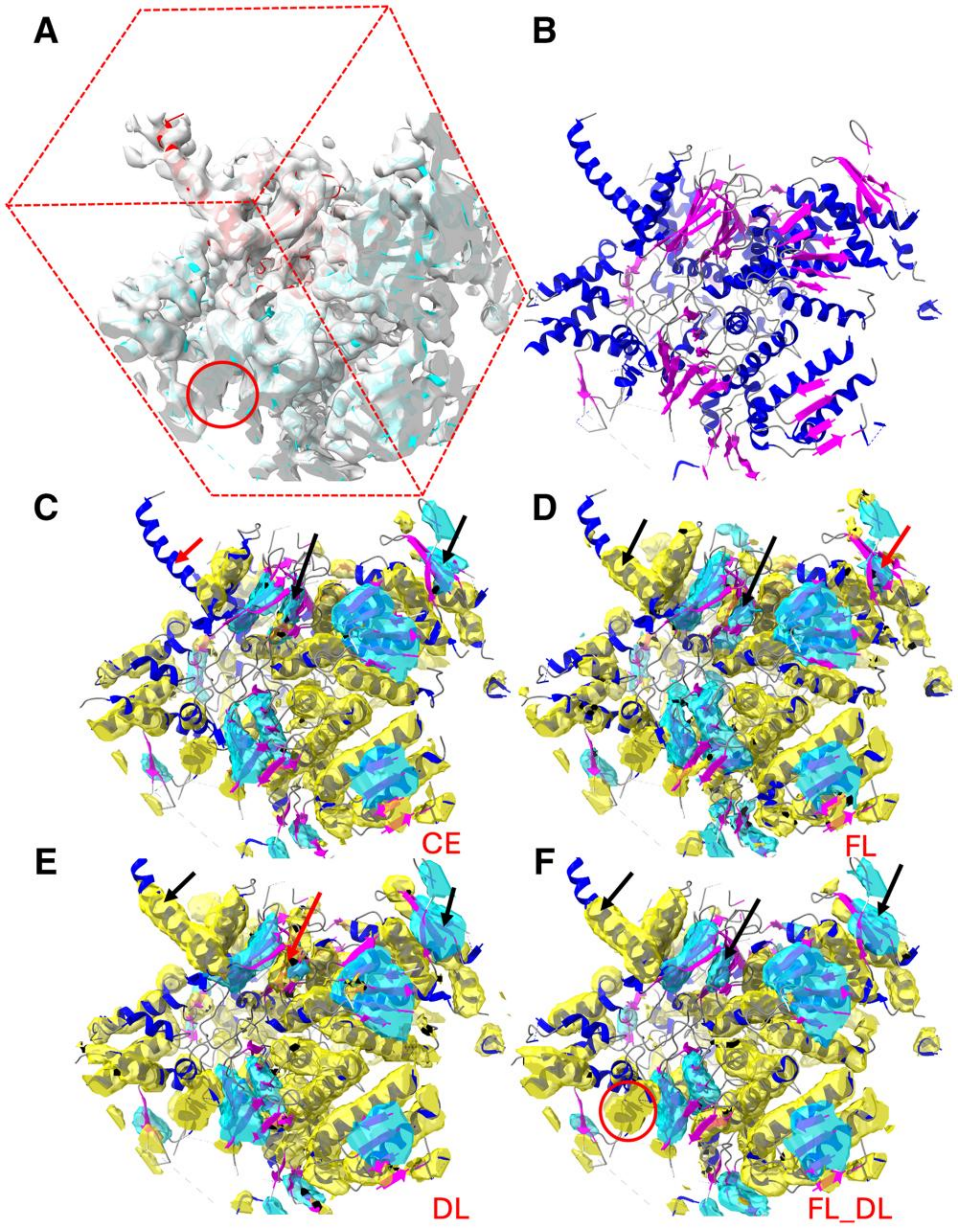
Segmentation of secondary-structure elements using CNN models trained with the CE, FL, DL, and FL_DL loss functions, respectively. (A) Center box of a cryo-EM density map (EMD-4089, gray) near the atomic structure of chain D of PDB-5LN3 (ribbon with the center chain D in red). (B) Atomic structure of the cropped region, indicating the helices (blue ribbon), β-sheets (magenta ribbon), and the remaining molecular segments (gray). (C) to (F) show the helix regions (yellow) and the β-sheet regions (cyan) that were detected using CE, FL, DL, and FL_DL, respectively (helices: blue ribbon; β-sheets: magenta ribbon). The red arrows point to the undetected or wrongly detected regions, and the black arrows, for the better-detected corresponding regions. The red circle indicates a region at the edge of the box in (A) and the corresponding detected partial helix in (F).

**Figure 3.**
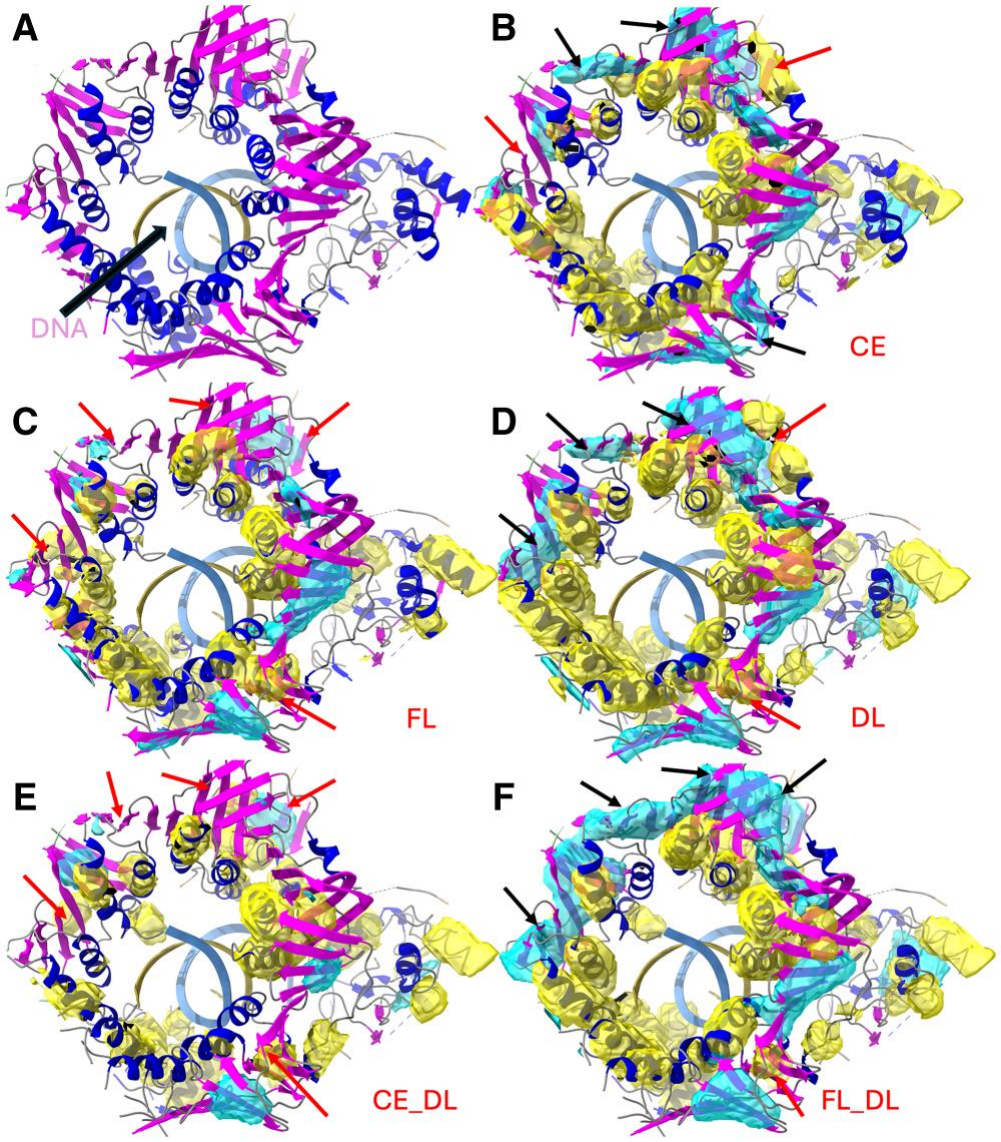
Detection of the helix region (yellow) and the β-sheet region (cyan) on the box-cropped cryo-EM density map (EMD-4141 cropped at chain B of PDB-5M1S). (A) Atomic structure (ribbon) on the corresponding cropped density map, indicating the helices (blue) and the β-sheets (magenta) of chain B of 5M1S (PDB-ID). (B) to (F) show the helix regions (yellow) and the β-sheet regions (cyan) that were detected via CNN models trained using CE, FL, DL, CE_DL, and FL_DL, respectively. The red arrows indicate the undetected or wrongly detected regions, and the black arrows, the better-detected corresponding regions.

Although our previous dataset represented a set of chain regions with good matching structures and low repetition, due to the masking, the trained model sometimes failed at the edge of a cropped map. Thus, end users were expected to prepare an input map without sharp edges, which was inconvenient for them. Therefore, in the present study, we added a step for generalizing the dataset into box-cropped pairs as follows. Each map/structure pair contains a complete central chain and partially cropped neighboring chains corresponding to a rectangular box whose dimensions exceed the central chain dimensions. For each pair, the density map region was cropped around the specified central chain using ChimeraX (Pettersen *et al.* 2021), and an additional 34 Å was cropped off from each dimension to provide the padding needed for the depth of field of our CNN architecture. For example, the box-cropped map (with padding) for EMD_4089-PDB_5LN3_D (Fig. 2) has a size of 112 × 104 × 128, and the box-cropped map for EMD_4141-PDB_5M1S_B (Fig. 3), 112 × 112 × 112. The dimension of the box-cropped cryo-EM map depends on the size of the chain. In this way, the data input to the CNN maximally preserve the center chain while providing the typical cropping artifacts that an end user would encounter. Because a box-cropped map contains an entire central chain and partial neighboring chains, the quality bin of the map was estimated using the quality of the central chain region of the map.

The secondary structure in the atomic structure was assigned using the Dictionary of Secondary Structure in Proteins (DSSP) (Kabsch and Sander 1983, Joosten *et al.* 2011). Residues were annotated as follows: residues with DSSP character codes H, G, and I were marked as *helices*; those with codes B and E were marked as *β-sheets*; and the remaining residues, including all nonprotein residues in the PDB entry, were marked as *backgrounds*.

Three classes were used to represent helix, β-sheet, and background voxels, respectively. Since the thickness of a helix or a β-sheet is generally within 6 Å, voxels within 3 Å from a Cα atom of a helix were labeled helix voxels, and voxels within 3 Å from a Cα atom of a β-sheet were labeled β-sheet voxels (Mu *et al.* 2021). The remaining voxels were labeled background voxels. They contained both nonmolecular voxels and voxels in loops, turns, and nonprotein (e.g. RNA or DNA) molecules.

### 2.2 Five loss functions

Five loss functions were implemented to study their effects—CE, FL, DL, combined CE and DL (CE_DL), and FL_DL.

Each input to the network is a 4D tensor, with the first dimension representing the total number of images in the mini-batch, and the remaining three dimensions representing the 3D density maps. Let *K* be the number of classes (in this case, K=3). Let *N* be the total number of maps in the training set, and $N^{i}$, the total number of voxels in the *i*th map, where $i\in \left\{1,\ldots ,\;N\right\}$. Let $y^{<i,j>}$ be the binary vector of length K that represents the true label of the *j*th voxel in the *i*th map, where $j\in \left\{1,\ldots ,\;N^{i}\right\}$. Since each voxel is assigned to only one of the three classes, only one of the K entries in $y^{<i,j>}$ is 1, and all the rest are 0 s. Let $p^{<i,j>}$ be the vector of ($p_{1}^{<i,j>},\;\cdots ,\;p_{K}^{<i,j>}$), where $p_{k}^{<i,j>}$ denotes the predicted probability of voxel <i, j> belonging to class k=1,…, K. Let $y=\left\{y^{<i,j>}\right\}$, and let $P=\left\{p^{<i,j>}\right\}$.

#### 2.2.1 Cross-entropy (CE), focal loss (FL), and dice loss (DL) functions

**Figure 4.**
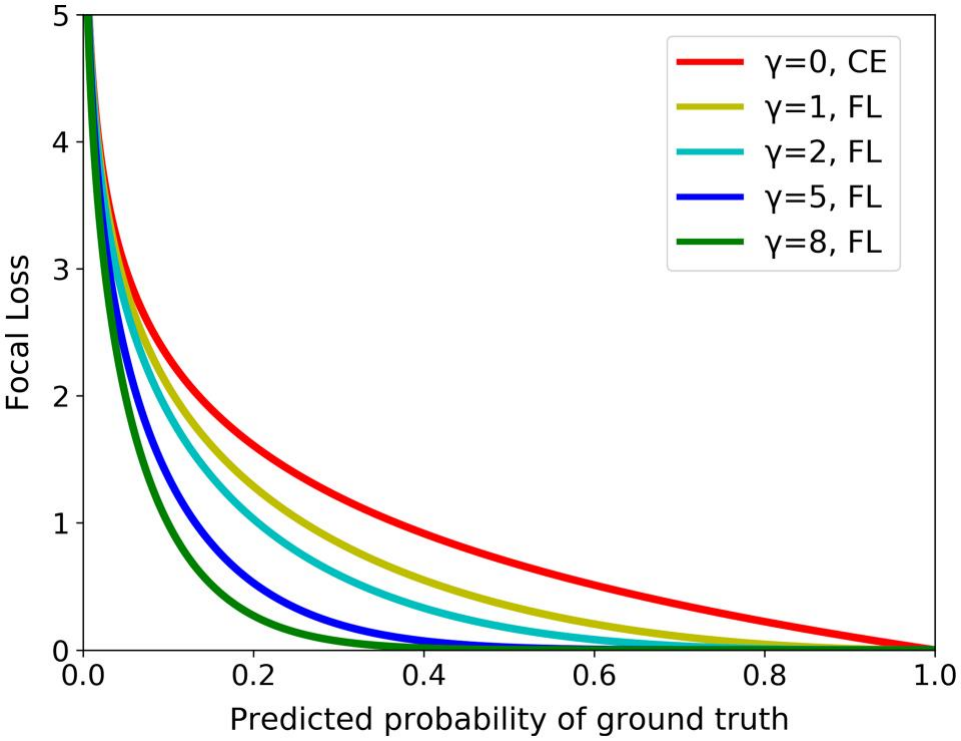
FL curves for the γ parameters used in this study. The FL measure was reduced to the CE measure when γ=0.

CE is a popular loss function in learning. It is defined in Equation (1). FL was designed to encourage the training to focus adaptively on harder examples that the current model poorly predicted (Lin *et al.* 2017). This was achieved by including the coefficient $({1-p_{t})}^{\gamma }$ in the standard CE loss, where γ≥0 is a hyperparameter and $p_{t}\in \left[0,1\right]$ is the predicted probability of a given example associated with the true label. FL is defined in Equation (2). As illustrated in Fig. 4, the larger γ was, the more the training was focused on harder examples. When γ = 0 FL was reduced to the standard CE. In this study, four values were explored for FL: γ = 1,2,5, and 8.

(1) $$\mathrm{CE}(y,P)=-\sum_{i=1}^{N}\sum_{j=1}^{N^{i}}\sum_{k=1}^{K}y_{k}^{<i,j>}l\mathrm{og}p_{k}^{<i,j>}$$

(2) $$\mathrm{FL}\left(y,P\right)=-\sum_{i=1}^{N}\sum_{j=1}^{N^{i}}{\left(1-p_{t}^{<i,j>}\right)}^{\gamma }{\log p}_{t}^{<i,j>}$$

Without loss of generality, let us assume that class K, or the last entry in $p^{<i,j>}$ (and $y^{<i,j>}$), represents the class for background voxels. The Dice coefficient is defined in Equation (3). With this definition, DL is defined in Equation (4).

(3) $$D\left(y,P\right)=2\sum_{i=1}^{N}\sum_{k=1}^{K-1}\frac{\sum_{j=1}^{N^{i}}y_{k}^{<i,j>}p_{k}^{<i,j>}}{\sum_{j=1}^{N^{i}}y_{k}^{<i,j>}+\sum_{j=1}^{N^{i}}p_{k}^{<i,j>}}$$

(4) $$\mathrm{DL}\left(y,P\right)=2-D(y,P)$$

#### 2.2.2 Combined loss functions: FL_DL and CE_DL

The combination of CE and DL (CE_DL) is popular in medical imaging (Taghanaki *et al.* 2019). When DL and CE losses are combined, as shown in Equation (5), the loss function reduces the effect of class imbalance.

FL can similarly be combined with DL (Zhu *et al.* 2019). The combined loss function FL_DL is defined in Equation (6). As in FL, four values were explored in the training: γ = 1,2,5,8.

(5) $$\mathrm{CE}\_\mathrm{DL}\left(y,P\right)=\mathrm{CE}\left(y,P\right)+\mathrm{DL}(y,P)$$

(6) $$\mathrm{FL}\_\mathrm{DL}\left(y,P\right)=\mathrm{FL}\left(y,P\right)+\mathrm{DL}\left(y,P\right)$$

### 2.3 Architecture, training, and validation

The CNN architecture had five composite layers, similar to DeepSSETracer (version 0.1). It was based on the 3D U-Net model (Çiçek *et al.* 2016) and used 4D tensors with different sizes as inputs (see Section 1.4 in the Supplementary Data for details). The input 3D maps were not complete EMDs (i.e. EMD IDs), unlike those used in earlier DL methods (He and Huang 2021, Wang *et al.* 2021); instead, we used box-cropped subregions to allow DeepSSETracer to focus the learning on locally interacting chains. The entire dataset, with 1355 cases, was split into a training set (1246 cases), a validation set (47 cases), and a test set (62 cases). The validation and test sets were randomly selected from the entire dataset to ensure that the training and test sets would have similar data distributions in different quality bins (Section 2.1). For example, among the 62 cases in the test set, 10 cases were in Bin 1, 29 cases were in Bin 2, and 23 cases were in Bin 3. To further investigate the effect of loss functions on other ways of splitting the entire dataset, we conducted a cross-validation experiment that involved four other splits of the data for training, validation, and testing (see Section 1.1 of the Supplementary Data).

For each loss function, training was conducted using the same training dataset. Three different learning rates: 0.001, 0.0001, and 0.00001 and four different γ values: 1, 2, 5, and 8 were explored. The optimal settings of the two hyperparameters were determined using the validation dataset. At each training iteration, the loss was calculated without the 34 Å padding of each dimension; and in the final resulting segmentation, the padding was removed due to the reduced confidence in the padding region. To select the best hyper-parameter among the different models, the F_1_ scores for helix and β-sheet detection were calculated for each case in the validation set, and the averages of the two scores for all of the cases were used to select the best hyperparameters for each of the five loss functions.

## 3 Results

We used the test dataset of 62 box-cropped cryo-EM density maps to investigate the performance of five models that were trained using the same data but five different loss functions: three individual (CE, FL, and DL) and two combined (CE_DL and FL_DL). Each cropped map contained the molecular mass of a center chain and partial neighboring chains. Each voxel of the map had a predicted label indicating one of the following three classes: helix, β-sheet, and other or background.

For each voxel on a test map, the predicted label was compared with the true label that was derived using the atomic structure as a reference. Since the depth of field of the designed U-net architecture was about 35 Å, the center box without the 34 Å padding was evaluated for the F_1_ score in each test case. The weighted F_1_ score was the average of all 62 F_1_ scores, with each chain’s contribution weighted by the number of helix or β-sheet voxels on each map. In other words, a chain with less helix content contributed less to the weighted average of F_1_ scores than a chain with more helix content. The residue-level F_1_ score was calculated using the predicted label at each voxel and the DSSP-annotated secondary structure residues. The predicted label of each residue was determined by voting on the voxels within 3 Å of the Cα atom of the residue. Only the residues in the center box were included in the calculation of the residue-level F_1_ score for the center box.

### 3.1 Performance of the three individual loss functions

CE and DL are popular loss functions in CNNs, each with its strengths and weaknesses. The concept of focal loss was recently proposed (Lin *et al.* 2017, Prencipe *et al.* 2022), with the intent of boosting the loss from difficult voxels. Compared to CE, the use of the focal factor in FL improved the F_1_-score for both helix and β-sheet classes, but more increase for β-sheet detection was observed, from F_1_ score of 39.9%–45.4% (Table 1). This suggests that the focal factor in FL particularly helps in the detection of β-sheets that are often harder to detect correctly than helices. As mentioned in the Introduction, irregularly shaped and low-abundance β-sheets present challenges in segmentation. In fact, our dataset currently contains a much lower content of β-sheet (13.46%) than that of helices (37.32%). Regardless of the reason for the difficulty in β-sheet detection, FL appears to serve the purpose of improving the accuracy for harder voxels. DL performs slightly worse than FL in helix detection. However, it has a noticeable difference in β-sheet class, with an F_1_-score of 43.4%, much higher than CE (39.9%). The difference between CE and DL may reflect the different designs of the functions to handle the background class. DL is focused on the helix and of β-sheet class, and it is less affected by class imbalance, usually from the background class.

**Table 1.** Weighted-average F_1_ scores of a 62-case test set for five loss functions used in training.

Loss function	γ	F1 helix (%)	F1 sheet (%)	F1 avg (%)
CE	NA	61.7	39.9	50.8
FL	2	**62.9**	45.4	54.2
DL	NA	61.8	43.4	52.6
CE_DL	NA	61.7	46.0	53.9
FL_DL	1	62.0	**48.7**	**55.4**

The best γ and F_1_ scores are shown. The highest F_1_ score in each category is highlighted in bold. More details for CE, FL, DL, and FL_DL are provided in Table 2.

Although FL, CE, and DL show an overall slightly different performance for most cases, their performances are much different in some cases. In the case of EMD-2026/PDB-4UJE chain BH (Fig. 1), the detection of β-sheet using CE, FL, and DL exhibits increasing F_1_ scores of 42.9%, 55.5%, and 61.2% (Table 2). When they are compared with the corresponding detection using FL (Fig. 1D), the three detected β-sheets using CE (cyan in Fig. 1C) appear to be all smaller than they should be. When DL, a β-sheet that was missed using CE and FL was partially detected (upper left arrow in Fig. 1C–E). The largest β-sheet at the center (cyan and center black arrow in Fig. 1E) was also detected more completely using DL than using CE or FL (Fig. 1C–E). Helix detection in this case also showed noticeable differences, with F_1_ scores of 57.1%, 60.8%, and 62.2% for CE, FL, and DL, respectively. One such difference is at the top center helix (blue ribbon in Fig. 1C–E) that was missed completely using CE, but not using FL and DL.

**Table 2.** F_1_-score evaluation of the detected helices and β-sheets in 62 box-cropped map/structure pairs for the CE, FL, DL, and FL_DL loss functions.

EMDB_PDB_Chain (resolution in Å)	Count (H/S/O)	F_1_		F_1_		F_1_		F_1_		Residue-level F_1_	
		CE (%)		FL (%)		DL (%)		FL_DL (%)		FL_DL (%)	
		H-V	S-V	H-V	S-V	H-V	S-V	H-V	S-V	H-R	S-R
0090_6GYK_M (5.1)	557/324/648	53.6	41.6	56.2	26.8	51.1	28.3	**57.9**	**48.8**	65.6	56.1
1657_4V5H_AE (5.8)	158/68/229	58.1	50.3	**61.7**	**53.5**	59.1	51.9	60.1	46.9	69.4	59.6
1657_4V5H_AM (5.8)	131/34/213	**59.7**	47.5	56.9	**54.4**	53.7	48.4	53.1	41.1	60.3	48.3
1798_4V5M_AE (7.8)	164/95/151	47.1	37.4	50.1	**48.3**	**50.7**	41.5	48.8	47.2	55.1	57.0
2422_4V8Z_BZ (6.6)	164/95/201	**60.4**	46.0	58.5	47.5	54.6	**60.0**	55.5	58.3	72.0	65.9
2594_4CR2_3 (7.7)	480/335/398	**65.5**	51.6	63.3	**58.2**	55.0	42.3	58.0	55.8	81.4	61.5
2594_4CR2_S (7.7)	786/33/189	66.7	0.4	64.9	1.4	**67.7**	**20.0**	67.5	0.7	82.1	0.0
2620_4UJE_BH (6.9)	213/86/161	57.1	42.9	60.8	55.5	62.2	61.2	**62.5**	**66.5**	77.3	76.2
2620_4UJE_CL (6.9)	322/91/372	55.3	28.5	60.5	40.8	**62.9**	32.7	62.1	**51.9**	72.4	68.0
2917_5AKA_O (5.7)	66/7/329	46.2	**12.0**	44.9	6.9	51.6	11.1	**52.3**	11.0	65.5	19.0
3491_5MDX_H (5.3)	296/6/149	59.7	0.0	**59.9**	0.0	54.0	0.0	56.9	**3.5**	63.8	0.0
3580_5MY1_I (7.6)	168/45/262	24.9	21.7	31.4	24.3	**36.0**	**36.2**	32.7	30.3	36.0	35.8
3581_5MYJ_AK (5.6)	75/27/124	**45.4**	54.3	42.2	57.4	42.1	**65.1**	3.4	51.3	0.0	65.6
3594_5N61_E (6.9)	380/78/259	50.1	4.9	46.4	14.3	**60.0**	22.8	51.0	**37.3**	59.7	45.1
3663_5NO4_H (5.16)	80/79/119	63.2	41.2	**63.8**	52.7	61.3	52.4	62.2	**54.3**	77.5	57.6
3850_5OQM_4 (5.8)	509/136/367	57.8	44.0	57.6	**53.0**	**60.4**	45.6	58.2	51.7	68.6	62.3
3850_5OQM_g (5.8)	468/126/231	73.9	42.4	74.6	36.7	70.2	48.0	**74.7**	**51.4**	91.8	59.1
3948_6ESG_B (5.4)	259/15/119	65.1	0.0	65.4	25.1	**69.9**	**34.0**	67.0	24.9	76.3	35.0
4041_5LDX_H (5.6)	775/49/647	70.6	53.4	**72.3**	**53.9**	69.4	46.9	70.9	46.3	85.7	57.1
4041_5LDX_I (5.6)	585/175/1015	67.8	46.7	**69.0**	**51.1**	65.5	46.4	68.3	48.3	82.8	55.9
4075_5LMP_Q (5.35)	71/59/70	**72.9**	45.9	67.4	59.1	71.5	**71.2**	72.7	65.7	82.4	76.6
4078_5LMS_D (5.1)	154/63/172	62.5	32.9	65.2	55.4	65.1	**57.4**	**70.2**	53.0	81.1	59.7
4089_5LN3_D (6.8)	582/214/453	67.6	54.1	66.2	56.5	65.3	56.0	**69.0**	**59.2**	79.4	63.8
4089_5LN3_G (6.8)	596/279/453	**70.9**	44.3	66.4	53.4	65.8	49.9	68.6	**54.1**	80.7	55.7
4100_5LQX_H (7.9)	282/65/326	**71.0**	55.0	68.6	61.0	65.9	**61.1**	61.0	58.1	74.3	72.4
4107_5LUF_M (9.1)	1116/10/253	45.1	**3.9**	**53.6**	0.3	44.8	1.9	46.9	1.3	58.7	2.1
4141_5M1S_B (6.7)	318/271/337	54.5	27.9	50.6	19.5	**58.5**	36.3	58.4	**44.7**	69.9	50.6
4177_6F38_A (6.7)	582/160/319	67.3	48.1	68.3	53.7	68.0	**59.6**	**69.2**	54.8	82.3	66.5
4182_6F42_G (5.5)	280/90/473	57.8	7.4	55.3	18.7	**58.4**	17.7	55.4	**28.1**	67.3	32.3
5030_4V68_B7 (6.4)	31/0/77	14.9	0.3	31.0	0.0	30.6	0.3	**32.8**	**1.9**	35.3	NA
5036_4V69_AD (6.7)	133/34/193	51.3	12.6	53.8	**46.4**	**55.5**	43.5	54	35.8	67.1	42.5
5942_3J6X_20 (6.1)	65/61/124	53.3	44.3	**54.9**	45.8	53.3	**51.5**	43.8	40.1	51.9	47.1
5942_3J6X_25 (6.1)	58/4/87	48.2	0.0	44.1	**35.5**	**57.0**	16.6	34.5	24.8	39.3	44.4
5942_3J6X_83 (6.1)	120/52/157	48.8	29.3	65.4	54.9	62.9	26.3	**68.5**	**57.0**	83.9	81.3
5943_3J6Y_80 (6.1)	34/28/76	62.7	54.9	61.2	**56.6**	**65.8**	43.6	54.6	54.9	59.1	65.3
6149_3J8G_W (5.0)	22/59/58	9.9	18.3	**29.5**	55.0	24.6	57.6	14.9	**61.3**	9.5	82.4
6446_3JBI_V (8.5)	414/134/213	57.7	27.2	**58.5**	37.8	55.8	**45.4**	53.7	41.9	72.6	55.0
6452_3JBO_AH (5.8)	210/157/264	67.6	**56.8**	68.6	51.9	69.2	48.8	**70.5**	49.7	81.1	56.4
6452_3JBO_AS (5.8)	358/103/398	46.5	24.8	61.2	**50.7**	**63.4**	26.7	61.7	38.2	67.1	58.1
6452_3JBO_AZ (5.8)	292/95/200	62.6	52.1	67.6	**60.5**	67.7	55.0	**67.7**	41.1	78.1	62.3
6452_3JBO_Ae (5.8)	162/66/186	54.4	33.1	**66.5**	**47.3**	65.1	31.9	66.0	37.6	75.9	43.5
6452_3JBO_C (5.8)	320/154/331	63.5	63.2	64.8	**67.9**	**65.6**	61.0	63.0	63.5	72.0	73.9
6456_3JBN_AL (6.7)	389/94/341	54.2	24.3	59.5	**44.9**	**60.1**	21.1	59.3	34.1	69.4	51.0
6456_3JBN_AP (6.7)	360/64/375	54.6	13.4	59.9	**27.5**	**62.6**	11.3	60.6	25.7	68.6	66.7
6456_3JBN_U (6.7)	442/203/390	70.3	**62.6**	**71.3**	61.8	69.3	53.8	69.9	57.6	81.9	65.1
6585_5IMR_p (5.9)	70/18/95	65.2	46.3	**66.6**	**56.6**	61.8	50.6	65.3	50.6	83.3	73.9
6585_5IMR_u (5.9)	46/6/38	33.1	35.2	**66.4**	25.3	65.0	**35.2**	54.2	22.8	60.0	44.4
6810_5Y5X_H (5.0)	254/12/188	51.0	2.7	61.0	5.8	51.0	**9.5**	**61.8**	7.9	73.9	11.3
7454_6D84_S (6.72)	334/43/127	49.2	22.8	43.9	**30.3**	**52.1**	27.3	44.3	29.5	50.4	40.0
8016_5GAR_O (6.4)	366/0/148	**60.0**	NA	59.6	NA	57.5	NA	55.6	NA	63.5	NA
8128_5J7Y_K (6.7)	965/17/271	**76.2**	51.1	74.7	58.1	73.3	38.6	73.9	**61.6**	89.3	85.1
8129_5J8K_AA (6.7)	664/95/572	61.8	41.9	**62.0**	**47.9**	61.4	36.2	60.4	45.0	73.3	53.5
8129_5J8K_D (6.7)	830/96/995	**60.6**	36.1	59.2	41.4	58.0	33.3	58.3	**45.2**	72.4	56.9
8130_5J4Z_B (5.8)	656/88/805	**74.2**	**58.7**	73.5	53.6	68.5	45.2	72.2	55.1	86.6	69.5
8135_5IYA_E (5.4)	319/91/256	62.8	29.1	61.7	**41.2**	**63.1**	36.4	63.0	34.8	73.4	38.5
8335_5T0H_K (6.8)	221/78/221	57.4	44.4	62.1	51.1	**64.4**	53.3	61.1	**54.8**	69.6	67.5
8357_5T4O_L (6.9)	273/107/458	**72.3**	**56.0**	71.0	52.9	66.7	52.6	65.5	55.5	78.6	67.5
8518_5U8S_2 (6.1)	612/240/840	62.9	48.3	**64.2**	**51.1**	61.2	48.2	61.7	48.0	78.6	56.3
8518_5U8S_A (6.1)	529/43/343	**76.2**	40.2	74.9	39.3	72.4	**44.8**	73.2	41.1	88.2	47.1
8621_5UZ4_J (5.8)	121/69/232	50.8	26.4	48.4	38.5	**53.8**	47.6	49.9	**50.1**	56.1	53.3
8693_5VIY_A (6.2)	323/299/485	58.4	28.3	62.2	43.1	57.7	40.2	**64.9**	**56.4**	80.5	67.0
9534_5GPN_Ae (5.4)	114/10/64	58.3	46.4	56.6	47.3	**59.5**	**52.4**	55.3	44.6	69.6	66.7
Weighted Average	20 724/5735/18 647	61.7	39.9	**62.9**	45.4	61.8	43.4	62.0	**48.7**	74.3	58.2

The center box of each cropped map indicates the EMDB ID, the PDB ID, the author-annotated chain ID, the resolution of the cryo-EM map, and the number of Cα atoms in the center box obtained from the author-annotated CIF file. Voxel-level F_1_-scores (in percent) are shown for helix and β-sheet detection in the center box when CE, FL, DL, and FL_DL were used to train the CNN models. In each case, the highest score for helix and β-sheet detection is highlighted in bold for each case. Residue-level F_1_-scores are shown for the FL_DL loss function. The weighted average of F_1_-scores over the 62 test cases was calculated using the number of voxels of helix/β-sheet/background in each class as weights. H, helix; S, β-sheet; O, other/background; NA, no voxel or residue existent (undefined F_1_-score).

The FL and DL detected regions are complementary on some occasions. For test case EMD-4089/PDB-5LN3 chain D, both CE and FL detected a β-sheet (center black arrow in Fig. 2C and D), but DL missed a significant part of the β-sheet (center red arrow in Fig. 2E). In another case EMD-4141/PDB-5M1S chain B (Fig. 3), helix detection shows a noticeable difference, with FL performing the worst among CE, FL, and DL (F_1_ scores of 54.5%, 50.6%, and 58.5%, respectively). Visual inspection showed that the difference was mostly due to the partial detection of multiple helices. The partial complement between CE/FL and DL makes it more advantageous to combine different loss functions in such cases.

### 3.2 Performance of the two combined loss functions

Each of the individual loss functions, CE, FL, and DL, had strengths and weaknesses. Since we observed complementary behavior between DL and CE or FL in some cases, we investigated two combined functions, CE_DL and FL_DL, using simple addition of the two individual loss functions. Both combined loss functions, FL_DL and CE_DL, improved the detection of β-sheets, compared to each of the three individual loss functions. The F_1_ scores for β-sheet detection were 48.7%, 46.0%, 45.4%, 43.4%, and 39.9% for FL_DL, CE_DL, FL, DL, and CE, respectively (Table 1). These suggest that among these five loss functions, FL_DL is the most robust for β-sheets. Moreover, its F_1_ score (48.7%) was higher than those of FL (45.4%) and DL (43.4%), confirming the benefit of using the combined function FL_DL for challenging voxels.

However, combining loss functions showed little benefit for helix detection. In this regard, the five loss functions showed similar overall performance levels, with F_1_ scores of 62.9%, 62.0%, 61.8%, 61.7%, and 61.7% for FL, FL_DL, DL, CE_DL, and CE, respectively (Table 1). The reduced benefit of combining loss functions for helix detection versus β-sheet detection was consistent with our interpretation that the combined loss functions were more effective for difficult cases, such as β-sheets, in our dataset. Since the five loss functions performed similarly in helix detection but FL_DL performed best in β-sheet detection, we recommend FL_DL as the most robust function to use in the CNN with the overall best performance.

### 3.3 Structural interpretation of the detected β-sheets using FL_DL

We investigated the effect of combined loss functions, particularly, the overall best-performing FL_DL, on three specific test cases. The three β-sheets (cyan, black arrows in Fig. 1F) appeared to have been detected more accurately using FL_DL than using CE, FL, or DL (red arrows in Fig. 1C and D, and the black arrows in Fig. 1E). In the case of EMD-2620/PDB-4UJE chain BH, CE and FL missed one β-sheet and detected a smaller portion of the other two, while DL detected all three, and FL_DL detected all three with better coverage. FL_DL had the highest F_1_ score for β-sheet detection (66.5%) compared to DL, FL, and CE (61.2%, 55.5%, and 42.9%, respectively; see Table 2). This case suggests that the following two situations can benefit from the use of the combined function: (1) when FL or DL misses a β-sheet (upper left arrow in Fig. 1D and E) whereas the combined loss function does not (Fig. 1F, upper left arrow), and (2) both FL and DL detect only parts of a β-sheet (two lower arrows in Fig. 1D and E) whereas the combined loss function detects a β-sheet more thoroughly (lower two arrows in Fig. 1F). In another test case, EMD-4141/PDB-5M1S chain B (Fig. 3), in which FL_DL performed best among the five loss functions in β-sheet detection, Scenario (2) was also observed. In that case, both DL and FL only partially detected two β-sheets (two left arrows in Fig. 3D and the corresponding area in Fig. 3C), but FL_DL detected both β-sheets more thoroughly (two left arrows in Fig. 3F).

Table 2 shows the details of the detection of helices and β-sheets in each of the 62 test cases. For each test case, the center box without the 34 Å padding was evaluated. For example, the center box of the box-cropped density map EMD-0090 at chain M of PDB-6GYK contained 557 helix Cα atoms, 324 β-sheet Cα atoms, and 648 other Cα atoms (row 1, column 2 of Table 2). This box-cropped density map contained much more protein than the masked chain M of PDB-6GYK that we used in an earlier study, which contained only 155 helix Cα atoms, 11 β-sheet Cα atoms, and 113 other Cα atoms (row 1 in Table 2) (Mu *et al.* 2022). In fact, the 62 test cases in the current study had a total of 20 724 helix residues; 5735 β-sheet residues; and 18 647 other residues, whereas the test set in our previous study had only 4700 helix residues; 1881 β-sheet residues; and 4718 other residues (Mu *et al.* 2022). Note that the number of residues in column 2 of Table 2 does not include any RNA and DNA atoms. Therefore, the actual molecular mass included in the current study was greater than that in the column. The 62-case test set in this study is a much larger test set than the masked-chain test set in our previous study, and it offered a robust validation of the performance of the loss functions shown in Table 2.

The different loss functions were compared at the voxel level, in which at each voxel, the predicted label was compared with the true label. For example, in the box-cropped EMD-4089 at chain D of PDB-5LN3 (Fig. 2), the helix detection exhibited voxel-based F_1_ scores of 67.6%, 66.2%, 65.3%, and 69% when CE, FL, DL, and FL_DL were used in the training, respectively, suggesting that in this case, FL_DL performed best in helix detection. The voxel-level F_1_ scores for β-sheets were 54.1%, 56.5%, 56%, and 59.2% when CE, FL, DL, and FL_DL were used in the training, respectively. CE_DL was omitted for brevity, since its overall scores were lower than those of FL_DL with an optimized γ (see Table 1). For the best-performing loss function, FL_DL, the residue-level F_1_ scores were calculated, for which the predicted label of a residue was determined by voting on the voxels within a 3 Å radius from the Cα atom of the residue. Since voting is a more robust measure of overall prediction at an amino acid, residue-level F_1_ scores are generally higher than voxel-level F_1_ scores. The averaged residue-level F_1_ scores for helix and β-sheet detection of all the 62 test cases were 74.3% and 58.2%, respectively—about 10%–12% higher than their voxel-level F_1_ scores for FL_DL (Table 2, last row).

### 3.4 DeepSSETracer tool incorporated in ChimeraX

**Figure 5.**
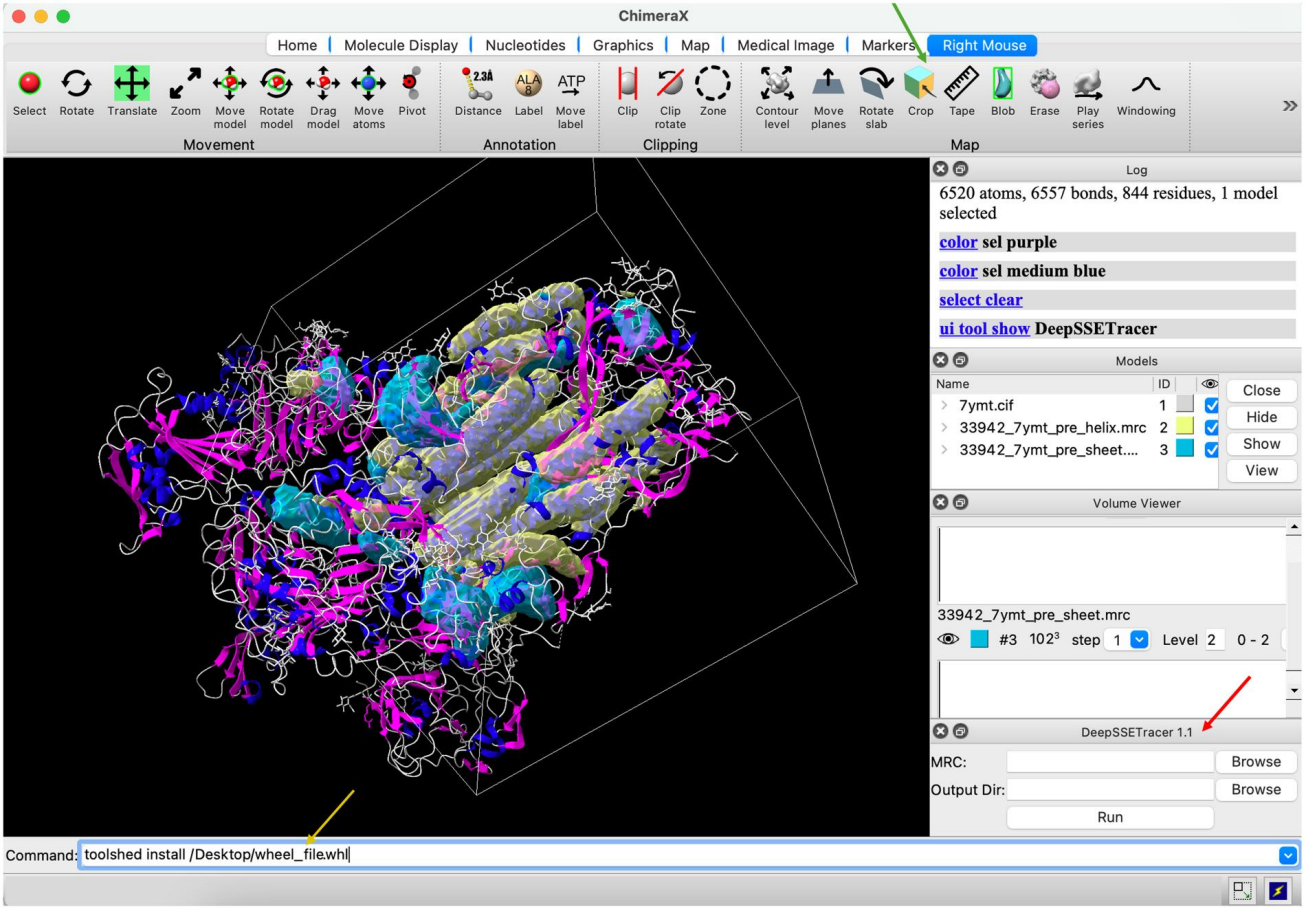
DeepSSETracer 1.1 integrated into ChimeraX. The rectangular subregion (white box) of the cryo-EM density map EMD-33942 was cropped using the crop function (green arrow) of ChimeraX. The helix (transparent yellow) and the β-sheet (transparent cyan) regions that were detected using DeepSSETracer 1.1 are superimposed on the atomic structure of the entire PDB entry (ribbon). The atomic structure is shaded blue (helix), magenta (β-sheet), and gray (remaining molecular segments). The DeepSSETracer 1.1 panel (red arrow) and the installation command (yellow arrow) are indicated.

DeepSSETracer is designed to target a subregion, instead of an entire cryo-EM map that is often much larger. It is also designed to run locally from a laptop without the need for computing clusters. The first version, DeepSSETracer 0.1, was introduced and integrated in ChimeraX (Mu *et al.* 2021). We developed DeepSSETracer 1.1 in this study and summarized in this subsection the main differences between the two versions. First, the box-cropped dataset used to develop the current model was much larger than the masked dataset used to develop the previous model. The current dataset had 1355 pairs that contained 1 889 687 residues, over 8 times the number of residues in the previous dataset. Second, the current data were more general than the previous data, since the current data were rectangular subregions of maps. This benefits end users, since rectangular subregions can be routinely created in a typical image-processing tool. Figure 5 shows the application of DeepSSETracer 1.1 in ChimeraX. The box-cropped region of the cryo-EM map was created using a mouse in the crop function of ChimeraX. Third, the model in DeepSSETracer 1.1 was trained using the FL_DL loss function, which demonstrated improved detection of β-sheets. The model in DeepSSETracer 0.1 was developed using a continuous learning training schedule and a weighted CE loss function. The model in version 1.1 appeared to be more robust than that in version 0.1.

## 4 Discussion

Our inspection of selected cases showed that FL and DL detected complementary regions in some cases, which explains why the combined loss function is beneficial. The interpretation of individual cases also showed that DNA or RNA can be distinguished as non-β-sheets and nonhelical voxels when they are not predominant on a map (Supplementary Fig. S1). However, adding a separate class to our current three protein-based classes may enhance the detection of both secondary structures of proteins and nucleic acids in future studies.

This investigation confirms our previous findings, which we obtained using a much smaller dataset (i.e. masked chains) (Mu *et al.* 2022). The content of the secondary structures in the two datasets also slightly differed. The helix residue content in the masked data was about 43%, which is higher than that in the current data (37%), presumably due to the higher loop/turn content of the neighboring chains, which were masked out in the earlier dataset. The β-sheet contents of the two datasets are almost the same (13%), even though the number of β-sheet residues in the current dataset is more than 8 times that in the masked dataset. Despite these differences in the total number of data and the secondary structure contents of the two datasets, we observed the following in both of them: (1) the FL_DL loss function detected β-sheets much better than did CE, and (2) the five loss functions had minimal overall differences in helix detection.

### 4.1 Data selection using cylindrical scores of helices

In this study, we used the data characterization and stratification method that we proposed in a previous study (Nguyen *et al.* 2023) to understand our current dataset. This dataset of 1355 map/structure pairs had about 37% helix residues and 13% β-sheet residues. Multiple factors might have contributed to the lower number of β-sheets in the dataset. First, there might have been more helix residues than β-sheet residues in the medium-resolution cryo-EM density maps in the downloaded EMDB entries. Second, our data requirement of having at least one helix in a chain might have eliminated the β-only chains. Unlike the characteristic cylindrical shape of a helix, the shape of a β-sheet varies depending on the β-sheet motif (common motifs are β-barrel, β-sandwich, β-prism, β-propeller, and β-helix), as well as the local environment and the local map resolution. Unfortunately, there is currently no reliable measure of the quality of a β-sheet’s match with the corresponding cryo-EM map. Therefore, we were forced to select suitable map/structure pairs for our training and test datasets based on the helix match quality. This limitation excluded pure β-sheet structures from our training and test sets. We expect the DL architecture to perform equally well on such pure β-sheet structures, but this still has to be tested. Thus, our present approach was validated only on maps that had at least one helix present in the crop box. In future studies, the development of a match quality measure tailored specifically to β-sheets would be desirable. This would allow us to include pure β-sheet test cases in both training and assessment, which could further advance the difficult β-sheet detection.

### 4.2 Cross-validation experiments

Table 1 shows the results of the training and testing for one split of our dataset. We conducted four additional experiments by creating four random splits (details in Section 1.1 of the Supplementary Data). In each of these four splits, each case in the testing set shared less than 35% sequence identity with any case in the training set when the center chain was considered. Since the center protein chain of a box-cropped image has a complete sequence, unlike most of its neighboring chains, we used the center chain sequence to conduct a sequence identity test. The four test sets were nonoverlapping and were created by randomly selecting sequence clusters with fewer than four members in each cluster. Although the performance of the five loss functions fluctuated across the five experiments or five splits of the dataset, the following were consistently observed. (1) Although the five loss functions hardly differed in helix detection, significant improvements in β-sheet detection were observed with the combined loss functions over any of the three individual functions. FL_DL and CE_DL had mean weighted average F_1_ scores of 47.0% and 46.2% for β-sheet detection, compared to 40.1%, 42.3%, and 43.3% for CE, FL, and DL, respectively (Supplementary Table S1). (2) FL performed better than CE in the β-sheet detection (42.3% versus 40.1%, respectively), confirming our earlier observation that FL is more advantageous for hard cases such as β-sheets. (3) FL_DL performed best among the five loss functions in β-sheet detection. In the cross-validation experiments, we observed less difference between FL_DL and CE_DL in β-sheet detection (47.0% versus 46.2%, respectively) than in Table 1; but in two of the five experiments, FL_DL performed slightly better than CE_DL. FL’s clear advantage over CE but FL_DL’s much weaker advantage over CE_DL could have been due to the domination of DL in the combined loss functions. In such functions, we observed that the DL was often much higher than the FL or the CE loss. Thus, further improvement may require another hyperparameter to weigh between the two loss terms in a combined function.

## 5 Conclusion

This is the first extensive study to evaluate the performance of an important neural network element in the segmentation of secondary-structure elements from medium-resolution cryo-EM maps. Unlike in the medical imaging field, in cryo-EM map applications, there is limited information on the performance of specific loss functions. We implemented three individual loss functions—CE, DL, and FL—as well as two combined functions and evaluated their performance using F_1_ score statistics. Our preliminary study, which had a smaller dataset, suggested performance differences among the five loss functions (Mu *et al.* 2022). The current study used a different dataset that contained a much larger volume of data and confirmed that the combined loss function FL_DL performed best overall in the detection of helices and β-sheets.

The hardest cases in secondary structure segmentation offer the greatest potential for future improvement. β-sheets are much harder to detect accurately in medium-resolution maps than helices. Aside from the natural challenge of the diverse shapes of β-sheets, the lower presence of β-sheet voxels in training data presents another challenge. In this study, we showed that these challenges can be addressed more effectively using FL_DL, as FL_DL detects β-sheets much better than CE, a popular loss function. In the five cross-validation experiments conducted, FL_DL showed a mean weighted F_1_ score for β-sheet detection of 47.0%, higher than that of DL (43.3%) and much higher than that of CE (40.1%).

Our results, using 62 map/structure test cases and the cross-validation experiments, show that individual FL and DL functions surpass the traditional CE function in β-sheet detection but perform similarly overall in helix detection. These results align with the intuition that FL boosts loss for more difficult voxels, such as for β-sheets than helices, and that DL is less affected by class imbalance and, therefore, helps the less-abundant β-sheet class.

We investigated specific cases to understand the behaviors of different loss functions. We found multiple instances where FL_DL more thoroughly detected a β-sheet compared to FL and DL. Sometimes, both FL and DL failed to detect a β-sheet that FL_DL detected thoroughly. In certain test cases, we suggested that the combined loss function can enhance detection accuracy through partial detection by either FL or DL.

Our results may provide insights into future improvements in the design of our network and training framework. The individual loss functions CE, DL, and FL exhibit unique properties in segmentation tasks. However, our analysis has shown that secondary-structure segmentation in medium-resolution cryo-EM maps requires properties at the intersection of focal loss and DL to deal with challenging voxels. Different loss functions can be easily combined, but future studies could try to fine-tune the combination. For example, FL and DL could be combined asymmetrically using weight parameters that would lean the combined function more toward either FL or DL. Moreover, to generate the greatest utility from a combined loss function, a nonlinear blending approach could be investigated. We expect such loss tuning, together with targeted β-sheet data selection, to yield further improvements in the future.

## Supplementary Material

vbae169_Supplementary_Data

## Data Availability

Testing data used in this work are available on request to the corresponding author. DeepSSETracer 1.1 is downloadable at https://www.cs.odu.edu/∼bioinfo/B2I_Tools/.
