# Supplementary material for: The combined focal loss and dice loss function improves the segmentation of beta-sheets in medium-resolution cryo-electron-microscopy density maps
Source: Bioinform Adv. 2024 Nov 22;4(1):vbae169. doi: 10.1093/bioadv/vbae169 (PMC11590252; doi:10.1093/bioadv/vbae169)
Supplement: vbae169_Supplementary_Data [file vbae169_supplementary_data.docx]

**Supplementary Material**

The Combined Focal Loss and Dice Loss Function Improves the Segmentation of Beta-Sheets in Medium-Resolution Cryo-Electron-Microscopy Density Maps

Yongcheng Mu^1^, Thu Nguyen^1^, Bryan Hawickhorst^1^, Willy Wriggers^2^, Jiangwen Sun^1^, and Jing He^1,*^

^1^Department of Computer Science, Old Dominion University–Norfolk, VA 23529, USA; ^2^Department of Mechanical and Aerospace Engineering, Old Dominion University–Norfolk, VA 23529, USA

*Corresponding author: Jing He, jhe@cs.odu.edu

- 1. **Creation of Cross-Validation Data Sets**

The cross-validation involved five data sets. In addition to the dataset used in the main text of the paper, four random splits of the entire dataset were created for the training, testing, and validation subsets (Xu and Liang 2001). To balance the need for fair sampling and for avoiding potential similarities between the testing data and the training data, the entire dataset was first clustered into 389 sequence clusters using the center protein chain of each rectangular box density map. Note that each entry in the dataset was composed of a 3D image and its corresponding atomic model. The boxed image was centered at a protein chain and included partial chains of its neighbors. Therefore, an image contained the complete sequence of the center chain but only partial sequences of its neighboring chains. A sequence cluster was created if the center chains had a sequence identity score of 35% or higher.

The creation of sequence clusters allowed us to avoid using potentially similar data in both training and testing, since if a member of a cluster is used in testing, no other members of the same cluster must be used in training or validation (Nguyen et al. 2023). Most of the sequence clusters had one to nine members. The largest cluster had 47 members, indicating the existence of a group with a large number of similar sequences for their center chains. For a test set to contain more cases from diverse chains (i.e., from different sequence clusters), smaller clusters with fewer than four members were considered candidate test data, since more clusters could be included in a test set. The four non-overlapping test sets were randomly created from those clusters with fewer than four members each; and if a cluster was used in a test set, all members of the cluster were included. Each of the four test sets included 60–61 map/structure pairs. After each test set was selected, the remaining data were split into the training and validation sets. For each validation set, we selected one chain from 50 random sequence clusters in the remaining clusters. Finally, the quality bin distributions were manually checked to ensure that they were balanced across these resulting subsets.

The training, model selection, and testing processes were the same for the five experiments. For each split, training was performed for each of the five loss functions (CE, FL, DL, CE_DL, and FL_DL). The same hyperparameters were sampled for the five experiments, except that the loss functions that involved FL had an additional hyperparameter: γ. The best-performing model for the validation set in each experiment was selected to perform the test. For all five splits, there were 1,246–1,248 cases in training, 47 in validation, and 60–62 in testing. For each experiment, the weighted-average F_1_ scores for helix and β-sheet detection for all of the test cases in a split were calculated. The weight of each test case was determined by the size of the helix or the β-sheet in each of the test cases, since the sizes of the helix or the β-sheet varied among the test cases. Simple averages of the helix F_1_ scores with the β-sheet F_1_ scores are provided in Table S1.

**Table S1.** F_1_-score evaluation for five experiments in the cross-validation. Experiments 1 to 4 used the random splits of the dataset described in 1.1. Experiment 5 was used and described in the main text of the paper. The weighted-average voxel-level F_1_ scores (in percentages) are shown for the detection of helices and β-sheets when the CE, FL, DL, CE_DL, and FL_DL loss functions were used in training, respectively, for the five splits of the data. Avg: simple average of the weighted-average F_1_ scores from the helix and β-sheet; Mean: the mean and SD: the standard deviation across the five experiments in each row. The highest mean F_1_-scores for the detection of helices and β-sheets are highlighted respectively.

| Loss Function | Structure | Avg_F_1__exp1 | Avg_F_1__exp2 | Avg_F_1__exp3 | Avg_F_1__exp4 | Avg_F_1__exp5 | Mean | SD |
| --- | --- | --- | --- | --- | --- | --- | --- | --- |
| CE | Helix | 62.2 | 64.7 | 60.6 | 63.5 | 61.7 | 62.5 | 1.59 |
|  | Sheet | 40.0 | 37.3 | 39.8 | 43.6 | 39.9 | 40.1 | 2.25 |
|  | Avg | 51.1 | 51.0 | 50.2 | 53.6 | 50.8 | 51.3 | 1.29 |
| FL | Helix | 62.7 | 64.7 | 60.4 | 63.0 | 62.9 | **62.7** | 1.53 |
|  | Sheet | 42.1 | 37.3 | 41.1 | 45.7 | 45.4 | 42.3 | 3.45 |
|  | Avg | 52.4 | 51.0 | 50.8 | 54.4 | 54.2 | 52.5 | 1.69 |
| DL | Helix | 60.7 | 63.2 | 59.5 | 62.4 | 61.8 | 61.5 | 1.45 |
|  | Sheet | 42.4 | 42.1 | 43.3 | 45.4 | 43.4 | 43.3 | 1.29 |
|  | Avg | 51.6 | 52.7 | 51.4 | 53.9 | 52.6 | 52.4 | 1.01 |
| CE_DL | Helix | 62.0 | 63.6 | 60.6 | 63.2 | 61.7 | 62.2 | 1.20 |
|  | Sheet | 45.4 | 44.1 | 47.6 | 47.9 | 46.0 | 46.2 | 1.58 |
|  | Avg | 53.7 | 53.9 | 54.1 | 55.6 | 53.9 | 54.2 | 0.76 |
| FL_DL | Helix | 62.0 | 63.6 | 60.6 | 63.5 | 62.0 | 62.3 | 1.24 |
|  | Sheet | 45.4 | 44.1 | 47.6 | 49.0 | 48.7 | **47.0** | 2.13 |
|  | Avg | 53.7 | 53.9 | 54.1 | 56.3 | 55.4 | 54.7 | 1.11 |

- 1. **Effect of Center-Chain Sequence Similarity between a Test Set and a Training Set**

In machine learning, different cases must be used for the testing and training data. In this application, the training and testing data were 3D images or rectangular boxed density maps. To avoid sharing data between the training and testing phases, deep learning methods, such as DeepSSETracer, EMNUSS, and EMap2Sec+, implement an indirect method of checking the similarity of chain sequences (He and Huang 2021; Mu et al. 2021; Wang et al. 2021). Suppose that a training image and testing image both contain 10 complete protein chains, their amino acid sequences are compared respectively to identify those sequences with high sequence identity. The assumption is that when the sequence identity is high, the two images are similar. However, this assumption ignores the geometric properties of an image and the relative geometric relationship among the protein chains. For example, one image may be an augmented version of another image, or the relative positioning of the individual chains may not be identical, even if some of the chains have high sequence identities. Therefore, using sequence identity to screen similarity between two images is an estimation method. However, no direct screening method has been implemented in secondary structure detection problems to date.

In this study, instead of using entire cryo-EM maps of EMDB as training data, we used box-cropped density maps of varying sizes centered at a specific protein chain. The box-cropped images produced partial chain sequences for most of the neighbors of the center chain. Sequence comparison is not reliable for partial chains since the partial chains correspond to ad hoc locations on the sequences. We implemented a sequence comparison for the center chains in any two images to estimate the similarity between the two images. However, the practical effect of the sequence identity between two center chains has not yet been analyzed. To examine the practical effect of center-chain sequence identity, we created two groups of test images. One group (Set_nonSim_Seq) included 26 box-cropped density maps, the center chain of each of which shared less than 35% sequence identity with any center chain in the training set. The other group (Set_Sim_Seq) contained 36 box-cropped density maps, the center chain of each of which shared more than 35% sequence identity with at least one center chain in the training set. The weighted-average F_1_ score of each set was calculated for the 26 images in Set_nonSim_Seq, and, similarly, for the 36 images in Set_Sim_Seq. We did not observe that the test set with center chains similar to some center chains in the training set always performed better than the test set with non-similar center chains, since the helix detection was worse overall for Set_Sim_Seq than for Set_nonSim_Seq (Table S2). The detection accuracy, in terms of the F_1_ scores, appeared to be related more to the quality of the image data than to the center-chain sequence similarity between the test and training data. It is likely that the determinant characters in the CNN network were more about local features than global features, which sequence similarity reflects on.

For Experiment 5, we used a set of 62 box-cropped map/structure pairs in the test set. The 62 test cases were composed of the 26 cases of Set_nonSim_Seq and the 36 cases of Set_Sim_Seq. Since we did not observe a systematic advantage of Set_Sim_Seq over Set_nonSim_Seq, we included all of the cases in the test set.

**Table S2.** Performance of two sets of test data with different center-chain sequence identities shared with the training set. Weighted averages of the F_1_ scores of Set_nonSim_Seq and Set_Sim_Seq for the detection of helices and β-sheets. Set_nonSim_Seq contained 26 test images, each center chain of which shared less than 35% sequence identity with any of the center chains in the training images. Set_Sim_Seq contained 36 test images, each center chain of which shared more than 35% sequence identity with at least one image in the training set. The performance of the five models trained using the CE, FL, DL, and FL_DL loss functions are shown.

| Loss | Set_nonSim_Seq F_1__H (%) | Set_Sim_Seq  F_1__H (%) | Set_nonSim_Seq  F_1__S (%) | Set_Sim_Seq  F_1__S (%) |
| --- | --- | --- | --- | --- |
| CE | 63.8 | 59.7 | 33.6 | 44.0 |
| FL | 63.8 | 62.0 | 40.3 | 48.8 |
| DL | 62.7 | 60.8 | 39.7 | 45.9 |
| CE_DL | 62.5 | 60.9 | 40.1 | 49.9 |
| FL_DL | 63.1 | 61.0 | 46.2 | 50.4 |

- 1. **Cases Containing Nucleic Acids**

Many cryo-EM density maps contain RNA or DNA. Although the design of our learning method does not have a separate “nucleic acid” class label, we observed that the trained models were able to distinguish nucleic acids from helices and β-sheets in many cases that contained them. For example, in EMD-2620/PDB-4UJE chain BH (Figure 1), the RNA region was clearly detected as a non-helix, non- β-sheet background for all five loss functions (Figure 1C–F). Similarly, the DNA region in EMD-4141/PDB-5M1S chain B (Figure 3) was marked as a background. Both cases had significant protein content, in addition to nucleic acids. However, in the case of EMD-5030/PDB-4V68 chain B7 (Figure S1), in which the molecular mass was predominantly composed of RNA, the detection wrongly detected parts of the RNA as β-sheets. This result suggests ****that the trained models are generally applicable to box-cropped density maps if nucleic acids do not dominate the 3D map.

**Figure S1.** Secondary structure segmentation for a box-cropped cryo-EM map dominated by RNA molecular mass. (A) The box-cropped cryo-EM density map EMD-5030 at chain B7 of PDB-4V68 superimposed on the atomic structure (ribbon). Helices (blue), β-sheets (magenta), and the remaining molecular segments (gray) were used to represent the atomic structure. (B) The detected helix regions (yellow) and β-sheet regions (cyan) superimposed on the atomic structure. Part of the RNA (wide gray ribbon) and one of the incorrectly detected β-sheets are indicated.

- 1. **3D U-Net Architecture in DeepSSETracer**

Table S3 shows the implementation details of the DeepSSETracer architecture. The network has five layers. Each Conv3D is followed by BatchNorm3D and ReLU, except for the output layer. The number of filters is provided in each convolution step (Table S3). The network has a total of 4,585,987 trainable parameters.

**Table S3.** Architecture details in DeepSSETracer. *p*: dropout rate; *k*: kernel size; *s*: stride. The parameters in Conv3D() and ConvTranspose3D(), from left to right, indicate the input channels, output channels, kernel size, stride, and padding.

| Layer ID | Type |
| --- | --- |
| Down_1 | Conv3D(1, 32, 3, 1, 1), BatchNorm3D, ReLU |
|  | Conv3D(32, 64, 3, 1, 1), BatchNorm3D, ReLU |
| Pool_1 | Dropout(p=0.5), MaxPool3D(k=2, s=2) |
| Down_2 | Conv3D(64, 64, 3, 1, 1), BatchNorm3D, ReLU |
|  | Conv3D(64, 128, 3, 1, 1), BatchNorm3D, ReLU |
| Pool_2 | Dropout(p=0.5), MaxPool3D(k=2, s=2) |
| Down_3 | Conv3D(128, 128, 3, 1, 1), BatchNorm3D, ReLU |
|  | Conv3D(128, 256, 3, 1, 1), BatchNorm3D, ReLU |
| Trans_1 | ConvTranspose3D(256, 256, 2, 2,0) |
| Up_1 | Conv3D(384, 128, 3, 1, 1), BatchNorm3D, ReLU |
|  | Conv3D(128, 128, 3, 1, 1), BatchNorm3D, ReLU |
|  | Dropout(p=0.5) |
| Trans_2 | ConvTranspose3D(128, 128, 2, 2, 0) |
| Up_2 | Conv3D(192, 64, 3, 1, 1), BatchNorm3D, ReLU |
|  | Conv3D(64, 64, 3, 1, 1), BatchNorm3D, ReLU |
|  | Dropout(p=0.5) |
| Out | Conv3D(64, 3, 1, 1, 0) |

References

He, J. and Huang, S.-Y. (2021) EMNUSS: a deep learning framework for secondary structure annotation in cryo-EM maps. *Briefings in Bioinformatics,* 22 (6).

Mu, Y. et al. (2021) A tool for segmentation of secondary structures in 3D cryo-EM density map components using deep convolutional neural networks. *Frontiers in Bioinformatics,* 1, 51.

Nguyen, T. et al. (2023) An approach to developing benchmark datasets for protein secondary structure segmentation from cryo-EM density maps. *In Proceedings of the 14th ACM International Conference on Bioinformatics, Computational Biology and Health Informatics.*, 8.

Wang, X. et al. (2021) Detecting protein and DNA/RNA structures in cryo-EM maps of intermediate resolution using deep learning *Nature Communications,* 12 (1), 1-9.

Xu, Q.-S. and Liang, Y.-Z. (2001) Monte Carlo cross validation. *Chemometrics and Intelligent Laboratory Systems,* 56 (1), 1-11.
